# Off-Axis Cavity-Enhanced Absorption Spectroscopy of ^14^NH_3_ in Air Using a Gain-Switched Frequency Comb at 1.514 μm

**DOI:** 10.3390/s19235217

**Published:** 2019-11-28

**Authors:** Satheesh Chandran, Albert A. Ruth, Eamonn P. Martin, Justin K. Alexander, Frank H. Peters, Prince M. Anandarajah

**Affiliations:** 1 Physics Department & Environmental Research Institute, University College Cork, Cork, Ireland; pmsc85@gmail.com; 2 School of Electronic Engineering, Dublin City University, Glasnevin, Dublin 9 D09 W6Y4, Ireland; eamonn.martin@dcu.ie (E.P.M.); prince.anandarajah@dcu.ie (P.M.A.); 3 Physics Department & Tyndall National Institute, University College Cork, Cork, Ireland; justinalexander101@gmail.com (J.K.A.); f.peters@ucc.ie (F.H.P.)

**Keywords:** cavity-enhanced absorption spectroscopy, off-axis alignment, frequency comb, gain switching, near infrared (IR) range, Fourier transform spectroscopy, NH_3_ sensing, ammonia detection

## Abstract

A custom-designed gain-switched frequency comb (GSFC) source was passively coupled to a medium finesse (*F* ≈ 522) cavity in off-axis configuration for the detection of ammonia (^14^NH_3_) in static dry air. The absorption of ammonia was detected in the near infrared spectral region between 6604 and 6607 cm^−1^ using a Fourier transform detection scheme. More than 30 lines of the GSFC output (free spectral range 2.5 GHz) overlapped with the strongest ro-vibrational ammonia absorption features in that spectral region. With the cavity in off-axis configuration, an NH_3_ detection limit of ∼3.7 ppmv in 20 s was accomplished in a laboratory environment. The experimental performance of the prototype spectrometer was characterized; advantages, drawbacks and the potential for future applications are discussed.

## 1. Introduction

The usefulness of the number of equally spaced, phase-coherent, narrow-band spectral lines of frequency combs (FC) has long been recognized for state-of-the-art applications in gas-phase laser (absorption) spectroscopy [1,2,3,4]. Mode-locked short pulse FC lasers can generate more than 10^5^ comb lines with very narrow bandwidth determined by the coherence time of the laser [5]. In many spectroscopic absorption experiments, it is challenging to exploit the narrow bandwidth of FC lines and their small free spectral range (FSR) because collisional or Doppler broadening mechanisms often limit the achievable resolution. Thus, FC lasers are frequently used in conjunction with dispersive spectrometers as broadband multi-line lasers (“spectral rulers”) where the resolution is then limited by the dispersion characteristics of the spectrometer [5]. In order to exploit the inherently high resolution of FC lasers, different detection schemes have been utilized, such as comb-cavity Vernier approaches [6], Fourier transform dual comb spectroscopy [7,8,9,10] and virtually imaged phase array detectors [11,12].

In contrast to the large number of comb lines generated by mode-locked short pulse FCs, gain-switched frequency combs (GSFC) only generate a small number of phase-coherent narrow bandwidth lines and span a much smaller spectral region (typically a few wavenumbers) [13]. Originally, GSFCs were developed for optical communication in the near infrared conventional (C)-band. The comb is generated by injecting polarization-controlled light from a master laser into a Fabry-Pérot slave laser and achieving gain switching by continuously driving the slave laser above and below its laser threshold using an amplified sinusoidal radio frequency (RF) signal. This creates a highly phase-correlated train of pulses corresponding to an optical comb in the frequency domain [13]. The technique enables tunability of the frequency comb’s center wavelength and of the free spectral range, also making it a suitable light source for trace gas detection. GSFCs are generally inexpensive in comparison to mode-locked femtosecond FCs, and they can be miniaturized through monolithically integrated device designs [13,14,15,16]. In 2016, GSFC-based dual-comb architecture was reported in an absorption study of H^13^CN in the 1.5 µm region [17]. More recently, a GSFC with an FSR of 10 GHz was employed for the first time in conjunction with a medium finesse *optical cavity* for the detection of H_2_S in the near IR range [18]. Using optical cavities to increase the optical interaction path length and, consequently, the absorption sensitivity, is a known powerful way to enhance the detection sensitivity of atmospheric trace species [19]. A number of publications on cavity-enhanced absorption spectroscopy (CEAS) with mode-locked short pulse FC lasers have demonstrated the potential of this approach [9,20,21]. The key challenge of cavity-enhanced FC absorption spectroscopy is to effectively couple the comb to the optical cavity by matching the narrow band comb lines with the mode structure of a high-finesse optical cavity. Different (more or less complex) methods for coupling and locking of FC lines to high finesse optical cavities have also been established [4]. 

In this work, we demonstrate that by passively coupling the light from a GSFC to an optical cavity in off-axis configuration, absorption spectra can be measured without active mode stabilization or locking. Using a non-confocal, symmetrical cavity (*g*_1_ = g_2_ =0.72) with off-axis beam alignment, the cavity’s mode structure becomes sufficiently dense to enable the passive coupling of the GSFC to the low finesse (*F* ≈ 522) cavity [22,23]. Since cavities in off-axis alignment are quite stable and less prone to misalignment, this approach significantly simplifies the application of GSFCs in the context of optical cavity applications. The reduction of complexity by eliminating any active mode matching schemes, the potential miniaturization of the GSFC (photonic integration) in conjunction with a robust cavity alignment scheme and the competitive cost of components represent ingredients for a compact, robust and hence commercially viable trace gas sensor for field application. 

In this proof-of-concept study, a GSFC with an FSR of 2.5 GHz was designed in a spectral region where significant absorption features of ammonia (^14^NH_3_) are located around 1514 nm. A stable prototype CEAS setup in off-axis configuration was established, and an ammonia detection sensitivity well below the legal exposure limit in a 20 s integration time was demonstrated (Section 3). Ammonia was chosen for this study owing to its high relevance in industrial and agricultural settings. NH_3_ is a hazardous and corrosive gas which increasingly causes air quality problems in the large-scale fertilization of agricultural land with manure and slurry. It occurs in the manufacturing of fertilizers and production of industrial refrigeration systems, and it is also an important chemical for synthesizing many pharmaceutical compounds and commercial cleaning products [24]. In nature, NH_3_ is emitted in volcanic eruptions, as well as in a series of bacterial and fungal decomposition processes [24]. It is also relevant for planetary atmospheres where Doppler-broadened lines can be used for temperature estimates [25] (and references therein). NH_3_ has a rich spectrum in the near-infrared range [25] (cf. Section 3.1). The chosen wavelength region around 1514 nm is suitable for NH_3_ detection because it contains absorption features with comparatively large absorption strengths (*S* ∼ 3.61 × 10^−21^ cm/molecule [25,26]), and other relevant trace gases (e.g., H_2_O) exhibit no or minimal absorption features around that wavelength which thus warrants a highly selective detection of ammonia.

The experiment and setup specifications are described in Section 2. The results and performance concerning NH_3_ detection are illustrated and discussed for single pass and cavity-based experiments in Section 3.

## 2. Experiment

**Figure 1:**
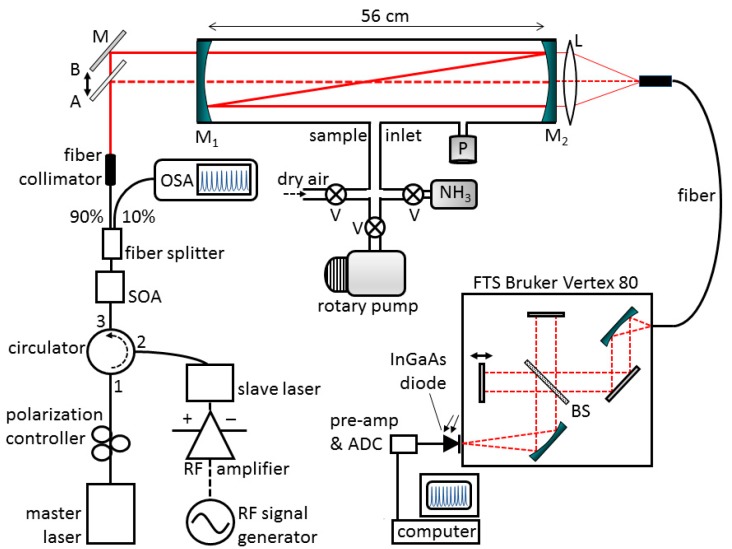
Schematic of the experimental setup. Mirrors M_1_ and M_2_ are high reflectivity dielectric mirrors (*R* ∼ 0.9940, diameter 1’’, plane-concave r = −2 m) forming an optically stable cavity with a length of 56 cm (effective number of passes ca. 167). The cavity could be replaced by a 661 cm single pass absorption cell. **M**: beam steering aluminium mirror mounted on a translational stage with a micrometer screw gauge. **L**: lens used for light collection. **P**: absolute pressure sensor, **V**: valves, **RF**: radio frequency (∼2.5 GHz), **ADC**: analog-to-digital converter, **SOA**: semiconductor optical amplifier, **OSA**: optical spectrum analyzer, **BS**: beam splitter.

A schematic of the experimental setup is shown in Figure 1. The main components of the GSFC source were a tunable master laser (Pure Photonics, PPCL550), a multimode Fabry-Pérot semiconductor slave laser (Eblana, EP1520-FP-H19-HM), a radio frequency (RF) signal generator (HP-83751A), a biased RF amplifier (Aldetec, AS01060), a polarization controller and a circulator. The GSFC configuration and signal detection scheme used here were similar to those reported previously [18]. In the current experiment, the GSFC was configured to generate ∼45 phase-coherent equally spaced comb lines with an FSR of 2.5 GHz, covering a range from ∼6604 to 6608 cm^−1^ (1514.23–1513.31 nm). Each comb line had an individual spectral bandwidth of ∼300 kHz [27]. The slave laser was injection locked at an absolute wavelength at 1513.827 nm, which was determined by means of a high-resolution optical spectrum analyzer (Yenista Optics, OSA20).

The quasi-continuous integrated output power of the current comb was ∼0.32 mW (–4.9 dBm). The maximum achievable output power scales with the comb’s FSR (i.e., the tunable frequency of the RF generator). Typically the power of the comb is determined by the repetition rate used for the gain-switching, in other words, the smaller the FSR, the smaller the achievable overall comb power [13]. Since in off-axis CEAS, a significant amount of light is lost, owing to predominant coupling of laser light to transverse cavity modes, the optical output power of the GSFC needed to be amplified. A semiconductor optical amplifier (SOA, Kamelian, OPB-13-0B-N-C-FA) was employed at port 3 of the circulator (see Figure 1) for amplifying the comb. The amplified integrated output power of the comb measured at port 3 was ∼6 mW (8 dBm). For monitoring the amplified GSFC emerging from the output port of the SOA, the light was split into two beams (ratio 90:10), and the less intense fraction of the beam was guided to an optical spectrum analyzer (OSA) for optimization purposes before experiments. The main beam (90%) was collimated (Thorlabs F260APC-1550) and guided to an optical cavity consisting of two plano-concave dielectric mirrors (diameter 25 mm, r = –200 cm, Layertec GmbH) separated by a distance of d = 560 ± 1 mm. The last beam steering mirror before the cavity was mounted on a translational stage with a micrometer screw gauge which was used to move the beam off the optical axis of the cavity (from position A to B in Figure 1). The reflectivity of the cavity mirrors was measured with a double-beam UV/Vis/NIR spectrometer (PerkinElmer, Lambda-1050). For on-axis alignment, a value of *R* ≈ 0.9960 was found; for ∼4 mm off-axis alignment (cf. Section 3.2), the value decreased to *R* ≈ 0.9940, and the error was estimated to be ±0.001. The optical cavity was enclosed by a stain-less steel vacuum tube (diameter ∼40 mm) which was fitted with access ports for evacuation and sample inlet. Before experiments, the cavity was always evacuated by a rotary pump to <0.1 mbar. The light exiting the cavity was collected with an achromatic lens and focused into a multimode light-guide (diameter 1.5 mm), which was connected to the entrance aperture of a Fourier transform spectrometer (FTS, Vertex 80 Bruker Ltd., with CaF_2_ beam splitter). In the FTS, the light was detected by an InGaAs photodiode; the best spectral resolution was 0.075 cm^−1^ using an integration time of 30 s (corresponding to the lowest available integration time of the FTS for this resolution). Generally, measurements were taken at 0.15 cm^−1^ resolution with an integration time of 20 s. Anhydrous ammonia (NH_3_, >99.5%) was purchased from Linde Nippon Sanso and used without further purification.

## 3. Results and Discussion

### 3.1. Single-Pass Absorption Spectrum of NH_3_

**Figure 2:**
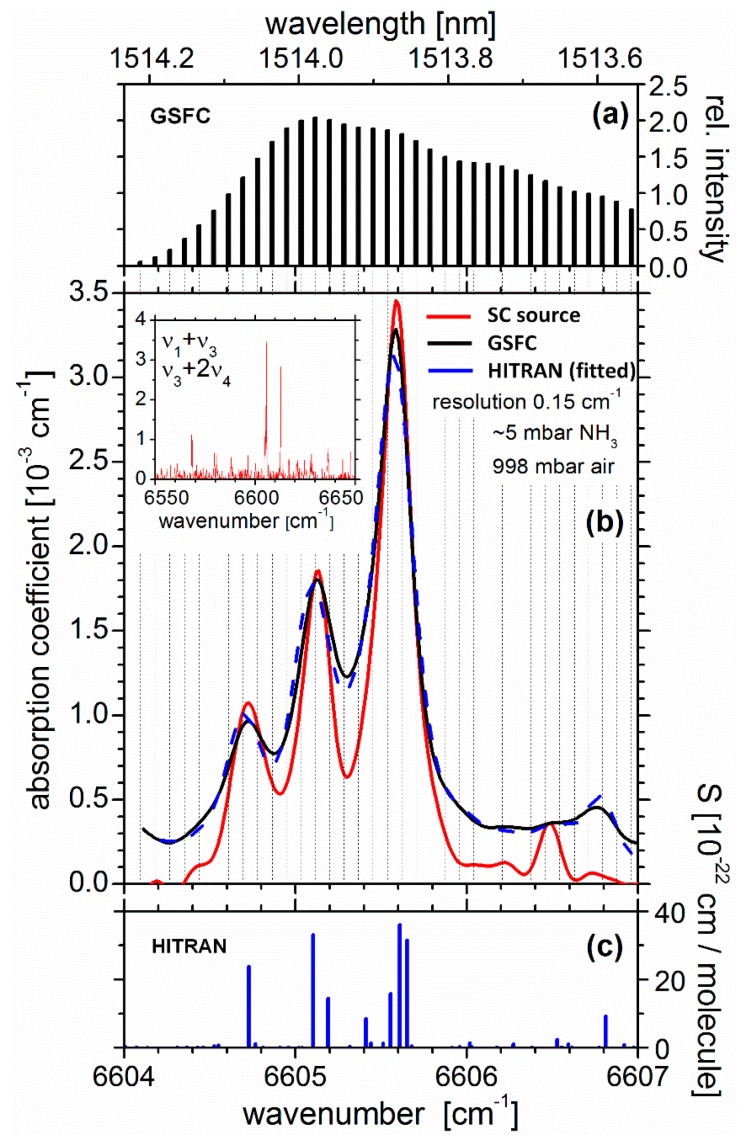
(**a**): Relative intensity stick spectrum of the GSFC (black trace) used for this single pass measurement. (**b**): Single pass absorption of NH_3_ of the same sample mixture measured with the GSFC (black trace) and SC source (red trace, expanded region of the inset) using a resolution of 0.15 cm^−1^ (Norton–Beer medium apodization). Vertical dashed lines correspond to the positions of the comb lines and illustrate the density of pivot points for the GSFC measurement. The NH_3_ mixing ratio of 5.0 ppTv was determined by fitting a HITRAN spectrum [32] for the given conditions to the absorption spectrum measured with the GSFC (dashed blue trace). (inset): Single pass overview absorption spectrum (6550–6650 cm^−1^) of static NH_3_ in 998 mbar of dry air measured using a path length of 661 cm and an integration time of 20 s. The strongest combination band transitions in this region belong to ν_1_ + ν_3_. (**c**): Stick spectrum of the absorption line strength as reported in HITRAN [26].

Before attempting to detect and quantify ammonia by cavity-enhanced absorption spectroscopy, the broadband overview spectrum of NH_3_ was recorded to identify and experimentally verify the positions of the strongest ro-vibrational absorption features in the region 6550–6650 cm^−1^ (∼1527–1504 nm; see inset in Figure 2b) and to tune the center wavelength of the GSFC to one of the strongest absorption lines of NH_3_ in that region. The spectrum was measured in a conventional single-pass absorption experiment using a *broadband supercontinuum laser* (Fianium SC450); the selected NH_3_ features used for detection are between ∼6604.2 and 6607 cm^−1^ as shown in Figure 2b. Conventional single pass absorption measurements were then taken in this narrow-band region with the *gain-switched frequency comb (GSFC)* in order to enable a quantitative comparison of conventional NH_3_ detection in single pass measurements with the off-axis CEAS approach discussed in Section 3.3. For the single pass measurements, the cavity in Figure 1 was replaced by a static gas cell of length, *d* = 6610 mm, with optical quartz windows at either end [28,29,30,31], using standard on-axis alignment (position A of the steering mirror in Figure 1). In most cases, the Fourier transform spectrometer was used to detect the light transmitted by the sample gas with an integration time of 20 s (one scan per spectrum using Norton-Beer apodization medium). All single pass measurements were performed by first filling dry air at approximately atmospheric pressure (∼1000 mbar) into the evacuated (∼10^–2^ mbar) gas cell, in order to measure the transmission, *I*_0_, without the sample species. Then, a small amount of NH_3_ was gradually leaked into the air-filled chamber, and the transmission, *I*, was measured. Owing to the reactivity and stickiness (high polarity) of NH_3_ and due to the fact that the gas cell was neither primed with NH_3_ nor long-term passivated prior to measurements, non-specific (wall) losses of NH_3_ occurred. The number density of NH_3_, *n*, was therefore determined by comparison with simulated HITRAN data [26] for relevant measurement conditions. All measured spectra were shifted by approximately –0.12 cm^−1^ to match the line positions with literature data. A HITRAN spectrum was calculated [32] based on the applicable resolution of the FTS (i.e., 0.15 cm^–1^), assuming an approximate number density of NH_3_ (e.g., based on the measured partial pressure) and the corresponding collisional air- and self-broadening, as well as Doppler broadening. This simulated HITRAN spectrum was then fitted to the measured ammonia absorption spectrum, *ε* (λ) ≈ [*I*_0_(λ)/*I*(λ)−1]*d*^–1^ *= n*σ + (a linear background) on the basis of a non-linear least square approach (minimum χ^2^) in the region between ∼6604 and 6607 cm^−1^ (blue trace in Figure 2b). This approach yielded a partial pressure of 5 mbar of NH_3_ (*n* = 1.32 × 10^17^ cm^−3^) for this measurement. The R-square value of the fit was 0.9918, and the 1σ standard deviation of the fit residuals (between 6604.2 and 6607.0 cm^−1^) was used as the minimum detectable absorption coefficient, $\varepsilon_{\min}^{\sin\mathrm{gle}}$= 2.93 × 10^−4^ cm^−1^Hz^−1/2^. On the basis of a 1:1 signal-to-noise ratio, the *single* pass detection limit for a 20 s acquisition time was hence estimated to be 106 ppmv (corresponding to $n_{\min}^{\sin\mathrm{gle}}$ = 2.76 × 10^15^ cm^−3^). This value (for a path length of 661 cm) is well below the lower explosion limit of 150 ppTv (parts per thousand by volume = 15% by volume). Figure 2b shows all relevant single pass spectra obtained including a broadband overview absorption spectrum in the inset as measured with the supercontinuum source. 

The ammonia spectrum has been extensively studied in the region between 6300 and 7000 cm^–1^ [25,33,34,35,36], where over 43 vibrational combination bands have been predicted by *ab initio* calculations [37]. The measured line positions in Figure 2b match the line positions reported in HITRAN (Figure 2c) within the instrument resolution [26]. There are ca. 1200 ro-vibrational transitions in the congested region 6550–6650 cm^−1^, shown in the inset of Figure 2b. The three ro-vibrational absorption bands between 6604 and 6606 cm^−1^ are the strongest features in that region and were thus used as a viable option to detect NH_3_. In the spectral region considered, not all transitions have been fully assigned. Ammonia is an oblate symmetric top molecule with a three-fold axis of symmetry (C_3v_ point group) and six normal modes of vibration: ν_1_(a_1_) symmetric stretch and ν_2_(a_1_) out-of-plane symmetric deformation (both parallel), ν_3_(e) anti-symmetric stretch and ν_4_(e) anti-symmetric bend (both perpendicular and doubly degenerate). The spectrum is subject to inversion doubling expressed in terms of the symmetry (*s* and *a*) with respect to the inversion plane. The strong ν_1_ + ν_3_ combination bands are centered around 6608.8 cm^−1^ (*s* type) and 6609.8 cm^−1^ (*a* type) [25]. The four strongest ro-vibrational transitions in the small detection window (all Δ*J* = 0) belong to the ν_1_+ν_3_ combination band [25] and have been assigned to RQ(1,0)*s* at 6604.7275 cm^−1^ (*S* = 2.384 × 10^−21^ cm/molecule), RQ(2,0)*a* at 6605.1042 cm^−1^ (*S* = 3.307 × 10^−21^ cm/molecule), RQ(5,0)*s* at 6605.5560 cm^−1^ (*S* = 1.577 × 10^−21^ cm/molecule) and RQ(3, 0)*s* at 6605.6088 cm^−1^ (*S* = 3.606 × 10^−21^ cm/molecule) [25]. Other overtone and combination bands that potentially also carry some intensity in the region considered here are, e.g., ν_3_ + 2ν_4_, ν_1_ + 2ν_4_ [25,33,34,35,36].

### 3.2. Off-Axis Coupling of the GSFC to the Cavity Without Target Species

**Figure 3:**
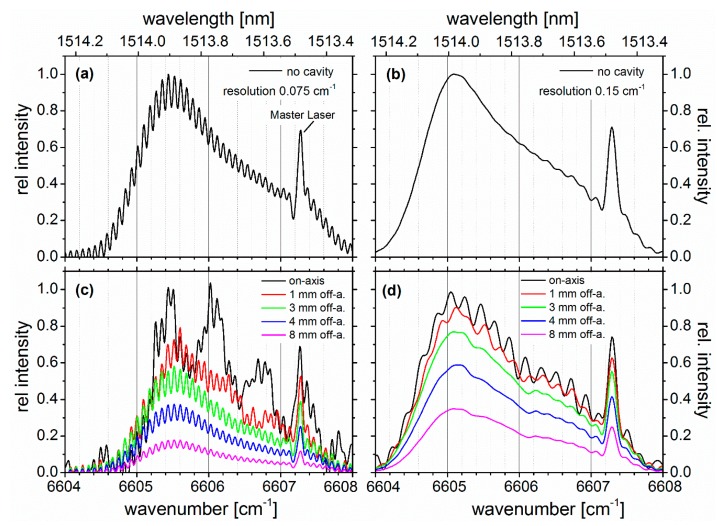
(**a**) and (**b**): Comb spectra measured before the cavity with a resolution of 0.075 cm^−1^ and 0.15 cm^−1^, respectively. (**c**) and (**d**): Relative intensity of the light transmitted by the cavity measured for different off axis-shift positions (in [mm]) of the steering mirror “M” (see Figure 1); see colour code. The resolution was 0.075 cm^−1^ and 0.15 cm^−1^ for panels (**c**) and (**d**), respectively. For these experiments, the cavity was filled with dry air (pressure ∼1000 mbar, static).

Following the single pass experiments, cavity-enhanced absorption measurements with the setup shown in Figure 1 were performed, using a cavity of 56 cm length, i.e., the mirror separation was more than 10 times shorter than the path length of the single pass gas cell. The characteristics of how the comb couples to the cavity was explored by gradually moving the GSFC beam away from the optical axis in a parallel configuration and measuring the cavity’s transmission spectrum within the high reflectivity range of the mirrors. Figure 3 shows the transmission spectra for two different resolutions (0.075 cm^−1^ in panels a and c, and 0.15 cm^−1^ in panels b and d) of the FTS (apodization; Norton–Beer medium), (Measurements at a resolution of 0.075 cm^−1^ in Figure 3 were taken with an integration time of 30 s under Norton–Beer (NB) weak apodization, as opposed to an integration time of 20 s and NB medium; i.e., the conditions for all other cases), measured for beam distances between 0 and 8 mm away from the optical axis of a cavity. It should be noted that the 1/e diameter of the GSFC beam was ≈1 mm. Hence, while the shift of the beam from on- to off-axis could be measured with accuracy (∼0.1 mm), the off-axis conditions cover a range of radii due to the physical size of the laser beam (see also error discussion in Section 3.3). The cavity was filled with dry air at 1000 mbar for these measurements. Upon moving the beam off-axis, the coupling efficiency (and thus the transmission) decreased as expected. However, due to the much denser mode structure experienced by the off-axis beam, the transmission spectrum went from a highly structured shape (black trace), due to interlaced comb/cavity-mode overlap, to a more even, unperturbed shape (magenta trace), which approached the comb spectrum before the cavity as shown for comparison in the upper panels a and b of Figure 3. For a cavity of 56 cm length built from spherical mirrors with a radius of curvature of 200 cm, the FSR was estimated to be ∼268 MHz; for an axially symmetric field distribution, the corresponding axial mode half-width was ∼342 kHz, which is similar to the half-width of the comb lines of ∼300 kHz [27]. Since the cavity’s geometry is far from confocal, for each axial mode there is a large number of transverse modes which “carry” a significant amount of light into the cavity. For on-axis alignment, there were approximately 9 axial cavity modes and the associated transverse modes in between two consecutive comb lines whose separation was 2.5 GHz (0.083 cm^−1^). Therefore, a non-uniform transmission spectrum was generated for on-axis comb beam alignment (black traces in Figure 3c,d). In off-axis configuration, the increased mode density caused each comb line to be in resonance with more modes, and mode overlap led to a quasi-uniform coupling of light to the cavity. Therefore, the mode structure constraint of the cavity gradually vanished by going from on-axis to an off-axis coupling arrangement, and the transmission spectrum gradually resembled the GSFC spectrum before the cavity, which is shown for comparison in the upper panels a and b of Figure 3 to illustrate this effect. (The significant intensity peak just below 6606 cm^−1^ is due to the master laser.) It can be compared to that of an optical low pass filter, which leads to the “smoothing” of the measured signal. This “optical smoothing” through off-axis configuration leads to an improvement of the signal-to-noise-ratio (SNR) in the cavity-enhanced absorption detection [22] (cf. Section 3.3).

### 3.3. GSFC Application to off-axis Cavity-Enhanced Detection of NH_3_ at 6604–6607 cm^−1^

The optimized GSFC was amplified using a semiconductor optical amplifier and coupled to the cavity as described in Section 3.2. The off-axis shift of the steering mirror (M in Figure 1) was set to ∼4 mm because the mode structure of the cavity is already insignificant for the transmission spectrum at that distance from the optical axis, and the intensity leaking from the cavity is higher than for larger off-axis distances (see Figure 3). Before experiments with ammonia, the cavity cell was always evacuated to a pressure < 0.1 mbar (including the inlet line and regulator valve), filled with dry air at ∼1000 mbar, and *I*_0_ was measured. For the off-axis CEAS measurements, the sensitivity of the setup was sufficient to detect vestiges of ammonia lingering in the regulator and valve’s volume (after the single-pass GSFC experiments). For the CEAS experiments, the ammonia bottle was thus not opened—only the reducer valve in the regulator was left open so that a small fraction of residual NH_3_ could escape into the inlet line. Ammonia leaking into the evacuated cavity from there was adjusted with a needle valve. After filling the evacuated cavity with small amounts of NH_3_ (at typical pressures of a fraction of a millibar), the static cavity was gradually filled with dry air to near atmospheric pressure (∼1000 mbar), and the transmission intensity, *I*, was recorded. 

**Figure 4:**
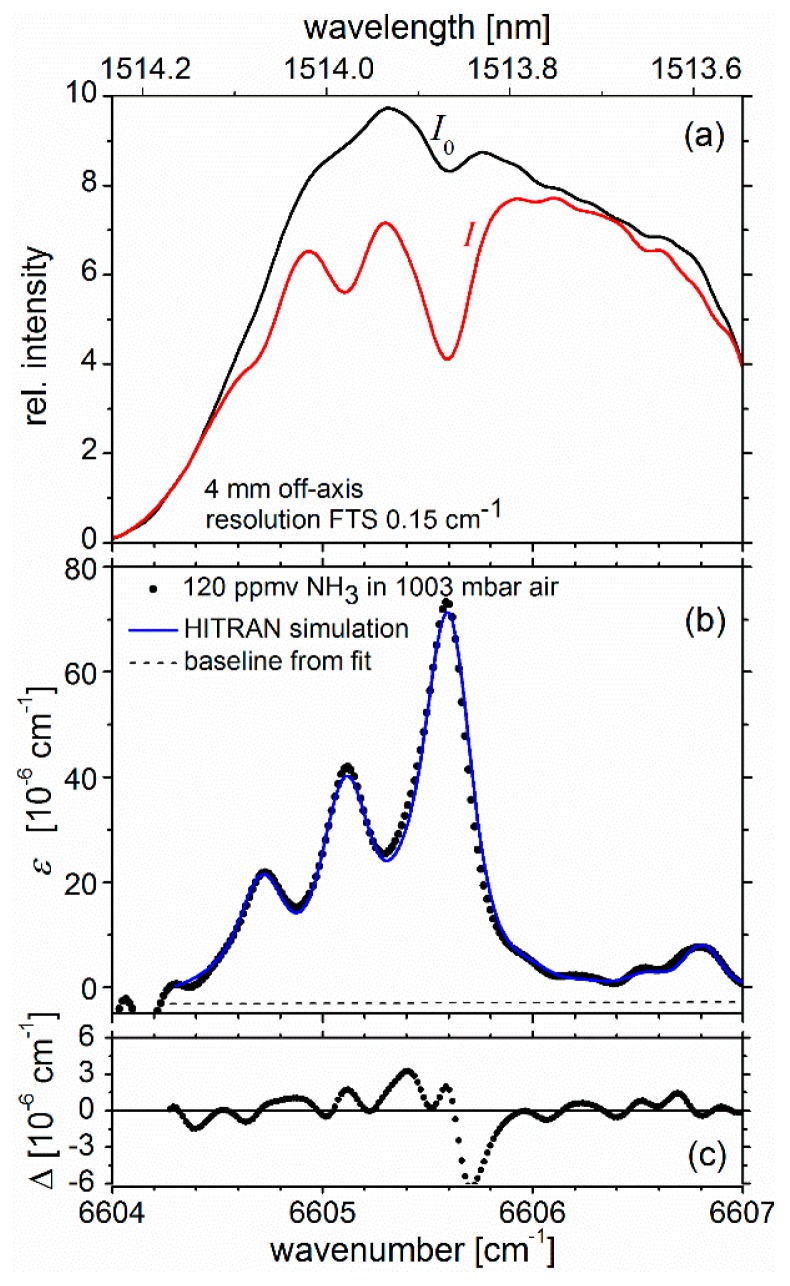
(**a**): Spectra of the relative intensities of light from the GSFC transmitted by the cavity for an off-axis distance of 4 mm from the optical axis of the cavity (spectral resolution 0.15 cm^−1^; apodization Norton–Beer medium). *I_0_*: Cavity filled with dry air (1003 mbar); *I*: cavity additionally filled with <0.2 mbar of static NH_3_. (**b**): Absorption coefficients (black dots) derived from data in the upper panel using Equation (1) (*d* = 56 cm, *R* = 0.9940). Simulated HITRAN reference spectrum of NH_3_ (blue line) fitted to the measured GSFC absorption spectrum using a nonlinear least square approach (min χ^2^) including a linear baseline (dashed line, slope (a1): 1.857 × 10^−7^, offset at 6604 cm^−1^: −4.755 × 10^−6^ cm^−1^). The wavenumber scale was shifted by −0.12 cm^−1^ to match the measured spectra and fitted HITRAN simulation. From the data, a number density of 180 ppmv was observed. (**c**): Absolute fit residuals, Δ = black dots − blue trace (interpolated); R-square value of fit 0.9926.

In total, 35 GSFC lines with a spacing of 2.5 GHz (∼0.087 cm^–1^) overlapped with the strongest absorption features at ∼6605.6 cm^−1^ (∼1513.9 nm). Even though the GSFC lines could be well resolved with the FTS at a resolution of 0.075 cm^−1^, a lower resolution of 0.15 cm^−1^ was used with Norton–Beer medium apodization because it was still sufficient to selectively detect ammonia under the current conditions and at the same time reduce the acquisition time to 20 s (cf. Figure 3). The spectra of *I*_0_(λ) and *I*(λ) (measured using dry air at ∼1000 mbar) are shown in Figure 4a. Figure 4b shows the measured absorption coefficient (black dots), ε, which was calculated based on Equation (1) [38]:

(1) $$\varepsilon \left(\lambda \right)=\left(\frac{I_{0}\left(\lambda \right)}{I\left(\lambda \right)}-1\right)\left(\frac{1-R\left(\lambda \right)}{d}\right).\;$$

(2) $$\varepsilon \left(\lambda \right)=n\sigma_{\mathrm{HITRAN}}\left(\lambda \right)+a_{0}+a_{1}\lambda$$

where *d* = 56.0 ± 0.1 cm is the mirror separation (static cavity, unpurged), and *R* = 0.9940 is the geometric mean of the reflectivity of the cavity mirrors (*R* was essentially constant across the wavenumber range shown). The number density of NH_3_, *n*, was evaluated to be 180 ppmv for the measurement shown, based on a non-linear least square fit (blue solid line in Figure 4b) of Equation (2) to the measured absorption coefficient. $\sigma_{\mathrm{HITRAN}}$(λ) is again a simulated HITRAN reference spectrum for approximate experimental conditions (partial pressure and temperature of NH_3_). Figure 4c shows the fit residuals, Δ, from which an $\varepsilon_{\min}^{\sin\mathrm{gle}}$∼1.04 × 10^−5^ cm^−1^ Hz^−1/2^ can be estimated corresponding to a 1σ detection limit of ∼3.7 ppmv for ammonia. This value is more than 5 times lower than the maximally allowable time averaged (8 hours) occupational exposure limit of 20 ppmv for ammonia [39,40]. (Indicative time-weighted averaged (TWA, 8 hours) limit according to the European directives 91/322/EEC, 2000/39/EC, 2006/15/EC, 2009/161/EU (12).) It is also well below the lower and upper explosion limits of 150 and 280 ppTv, respectively [41]. 

The 1σ cavity-enhanced detection limit turned out to be smaller than anticipated from the single-pass measurement as shown in the previous section. Based on the effective absorption path length of ∼93.3 m, which is 14.1 times longer than in the single-pass experiment. Thus, the anticipated limit of detection in 20 s (assuming no loss of signal-to-noise in the intensity measurement) would have been 7.5 ppmv (=106 ppmv/14.1). Thus, the value found was approximately a factor of ∼2 better than that predicted from the single-pass measurement. The reason for this discrepancy is not fully understood and is likely due to the poorer fit of the single-pass experiment from which the detection limits were derived. (The R-square coefficients of determination for the fit of HITRAN spectra to the data of the single pass and cavity measurements were 0.9918 and 0.9926, respectively.) Even though the conditions between the single-pass and cavity experiments were comparable, the full potential of the single-pass experiment was probably not reached owing to a non-optimum alignment of the FT spectrometer.

### 3.4. Uncertainties and Stability Considerations

The off-axis cavity-enhanced absorption measurement is affected by systematic errors, which are inherent to the setup and are discussed here in turn:

1. One of the smallest systematic uncertainties is the measured cavity length which was estimated to be Δ*d* = ±1 mm (≈0.2%).
2. Another general systematic error is caused by the dependence of the mirrors’ reflectivity on the shift of the laser beam away from the center of the mirror. The reflectivity at the center of the cavity mirror was measured with an absorption spectrometer to be *R* = 0.9960 for normal incidence. At an offset from the mirror center of ≈4 mm, the reflectivity was found to be *R* = 0.9940. Due to the finite diameter of the beam in the reflectivity measurement and also in the CEAS measurements with off-axis alignment (GSFC beam diameter ∼1 mm), the uncertainty Δ*R* was estimated to be ±5 × 10^−4^. This uncertainty results in an error of Δ(1−*R*) ≈ 8.3%, which is relevant for the evaluation of the absorption coefficient. Based on this systematic error alone, absolute gas concentrations are affected, causing an error in the detection limit of approximately ±0.3 ppmv.
3. A systematic uncertainty is also caused by the small inherent intensity noise of the GSFC coupling to the cavity in on- and off-axis configuration, which was estimated by recording 50 consecutive transmission spectra (30 s integration time) of the cavity at intervals of 40 s. At each wavenumber in the spectrum, the standard deviation of the 50 measurements was evaluated for on- and off-axis (shift of 4 mm) measurements. The mean 1σ standard deviations,${\bar{I}}_{\mathrm{SD}}$, averaged over all wavenumbers for on- and off-axis configurations were found to be 0.042 (=4.2%) and 0.0014 (=0.14%), respectively. The maximum values of standard deviation, $I_{\mathrm{SD},\max}$, in on- and off-axis configuration were 0.3331 (=33.3%) at 6604.74 cm ^−1^ and 0.005 (=0.5%) at 6604.78 cm^−1^, respectively. Assuming the latter maximum deviation as the sole systematic error concerning the intensity (Δ*I =* 0.5%), an optimal (*theoretically achievable*) lower limit of the minimum detectable absorption coefficient of 5.4 ×10^−7^ cm^−1^ for an integration time of 20 s can be estimated. This corresponds to a detection limit of 830 ppbv, i.e., approximately a factor of 4.5 below the detection limit evaluated from the absorption measurement of NH_3_ used here to determine the *experimental* detection limit.
4. The absorption cross-sections as well as the air- and self-broadening parameters in the HITRAN database have uncertainties that add to the overall absolute systematic error of the calculated absorption coefficients (and number densities). The five strongest absorption features of NH_3_ in the region between 6604 and 6607 cm^−1^ are given in Table S1 (supplementary material), together with the relative uncertainties of the corresponding absorption strengths (Δ*S*), all of which are below 10%. For all other (weaker) absorption features in the relevant region, the uncertainties are generally larger than 2% and smaller than 20% [26]. The maximum relative uncertainty of the self- and air-broadening coefficients for the absorption features at 6604.728, 6605.104 and 6605.609 cm^−1^ is 5%, for features at 6605.190 and 6605.652 cm^−1^, it is 20% [26]. As a conservative estimate for the overall systematic error, Δ*S*_overall_, arising from these uncertainties, we simply used the sum of the maximum uncertainty of the strongest feature at 6605.609 cm^−1^ (10%) and the average of its line broadening uncertainties (5%); i.e., Δ*S*_NH3_ = 15%. Finally, the calculated HITRAN reference spectrum was also calculated for approximate conditions (in terms of partial pressures and temperature for Doppler broadening) and was only linearly scaled (with background correction) to the measured data in the least-square fit to the measured spectrum, which leads to a small systematic discrepancy, which is treated as negligible.

Based on the considerations of errors (1)–(4) of experimental parameters, it is obvious that the uncertainty of the spectroscopic data on the NH_3_ transitions dominate the systematic error of the measured number density, followed by the uncertainty in the reflectivity. The overall absolute uncertainty of the number densities was estimated from Gaussian error propagation to be Δ*n* = ± 0.64 ppmv (=17.3%).

Finally, the measurements were quite reproducible, and by changing the NH_3_ concentrations by almost one order of magnitude, linear absorption behavior was observed (this is illustrated in Figure S1 in the supplementary material).

### 3.5. Selectivity in the Near IR range and Potentially Interfering Species

**Figure 5:**
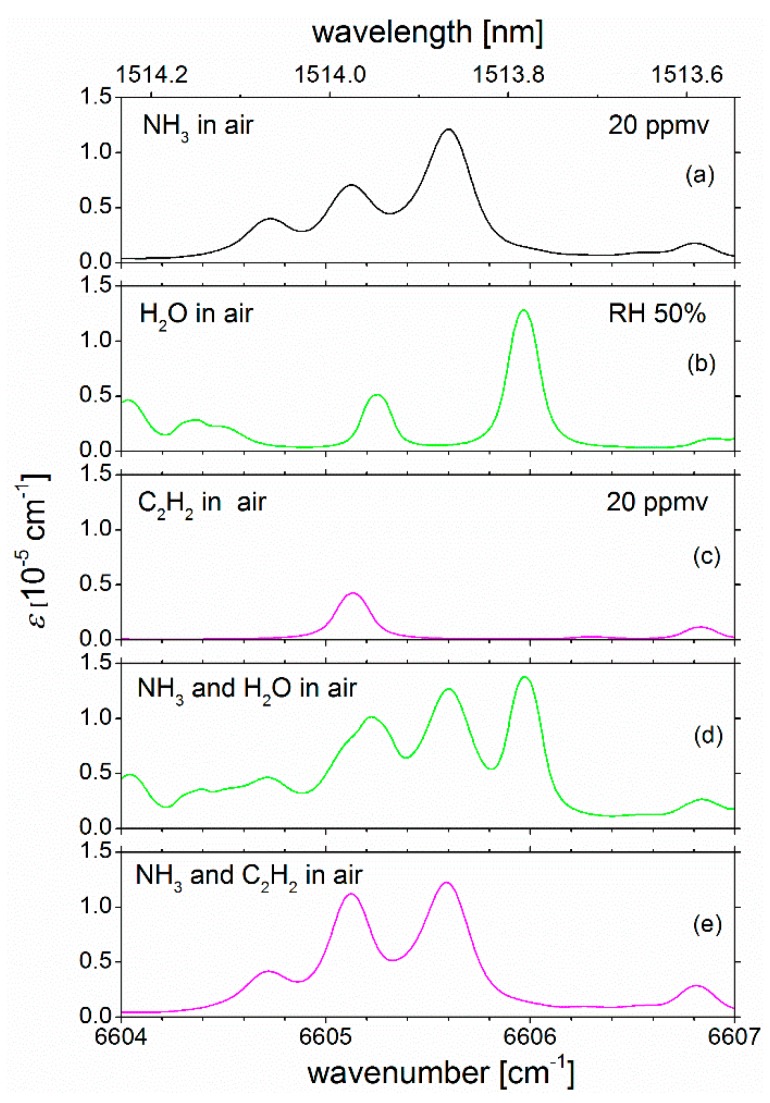
HITRAN simulations of absorption spectra (resolution of 0.15 cm^−1^) of (**a**) NH_3_ (at the occupational exposure limit of 20 ppmv), (**b**) H_2_O at a relative humidity (RH) of 50%, and (**c**) C_2_H_2_ (at 20 ppmv), all in 1 atm of air. Panel (**d**) shows a simulation for NH_3_ (20 ppmv) and H _2_O (RH 50%). Panel (**e**) was calculated for NH_3_ and C_2_H_2_, both at 20 ppmv. The figure illustrates the sufficient selectivity for NH_3_ at the resolution used in this work. By fitting several reference spectra in Equation (2), NH_3_ mixing ratios can still be retrieved with confidence.

It should be noted that the transitions for the detection for NH_3_ were chosen because they allow for good selectivity for NH_3_ within the C- and S-bands in the near IR range. In other words, other common gases with overtone combination bands in this spectral region generally have significantly smaller absorption strength than NH_3_ according to HITRAN (a brief list of potential interfering species in the spectral region between 6604 and 6607 cm^−1^ is stated in Table S1 (supplementary material)). Table S1 illustrates, that, with exception of acetylene (C_2_H_2_), some of the most common gases have significantly weaker absorbance than NH_3_; thus, their absorption generally does not need to be considered in fitting absorption coefficients according to Equation (2) to measured spectra. The only exception in this regard is potentially water. Due to its large abundance in many environments water may need to be included in the spectral fitting depending on the humidity of the gas sample. Even though in the current measurements the sample air was dry enough for water to not play a role, there are several weak absorption lines in the spectral region used here, which need to be considered in the evaluation of the spectra. To illustrate this aspect, spectra of NH_3_ were simulated in the presence and absence of water (also for acetylene, which would have to be present at a similar mixing ratio as NH_3_ in order to play an “interfering” role in the C- or S- band). Figure 5 shows calculated HITRAN spectra of NH_3_ at 20 ppmv (=occupational exposure limit) with and without spectral features of water at a relative humidity (RH) of 50% (Figure 5a,b,d). A comparison with C_2_H_2_ absorption features at 20 ppmv is also shown in Figure 5c,e. The simulations were carried out assuming a spectral resolution of 0.15 cm^−1^ in agreement with the experimental resolution used here. The water absorption features are distributed over the spectral range in such a way that retrieval of the data and hence selectivity will not be affected. If the correct reference spectrum of water is included in the fit, NH_3_ can still be retrieved. For C_2_H_2_, it is interesting to note that two out of three absorption bands overlap largely with those of ammonia. Figure 5 illustrates that the full range, i.e., 6604−6607 cm^−1^ will have to be used for the data retrieval. A situation where the ratio of water over ammonia concentration is so large that ammonia cannot be retrieved anymore was not found down to the stated detection limit of 3.7 ppmv. Even for NH_3_ mixing ratios at the detection limit and a water concentrations corresponding to RH 90% (see Figure S2, supplementary material), the ammonia features are still visible in the spectrum, and retrieving mixing ratios from a fit over the 3 cm^−1^ range should be possible (please compare Figure 4b in Varma et al. [42]).

### 3.6. Benchmarking the NH_3_ Detection in the Near IR Range

A general review of different types of NH_3_ sensors (catalytic, metal-oxide, optical) and their sensitivity requirements and performances in various sensing environments and applications was given by Timmer et al. [43]. For spectroscopic trace gas detection, the near infra-red is known to be an unfavourable regime because absorption cross-sections are generally small due to strong Franck–Condon inhibition in that spectral region. The advantages of working in the near IR range are, however, sufficiently narrow absorption features and hence good achievable selectivity, low scattering efficiencies and the availability of affordable cutting-edge optical technology.

In this study, the sensitivity was mostly limited by the use of a low finesse cavity. The effective path length in the cavity with modest mirror reflectivity (*R* = 0.994) was merely ∼93 m, which is within the same order of magnitude of absorption path lengths of some multipass cells [44] in the near IR range reported in the literature. In Refs. [45,46], a sensitivity of 0.7 ppmv in 1 min for the detection of NH_3_ at 6528.8 cm^−1^ (1531.7 nm) was reported using a diode laser based multipass (Herriott) setup with an optical path length of 36 m. Several cavity-enhanced detection methods have also been applied to NH_3_ in the near IR range. Peeters et al. [47] reported an open-path CEAS setup (mirror reflectivity of 0.9997) of NH_3_ with an approximate detection limit of 0.1 ppmv in 1 s, employing a tunable external cavity diode laser operating at 6568.3 cm^−1^ (1522.5 nm) [47]. A mode-locked frequency comb in combination with a high finesse optical cavity (*R* = 0.99989) was used for the detection of NH_3_ in human breath, exhibiting an estimated sensitivity of 18 ppbv in 30 s around 1512.2 nm [48]. One off-axis cavity enhanced detection setup for NH_3_ has also been reported by Manne et al. [49], but in the mid-IR range at 970 cm^−1^ (10.309 μm) using a pulsed quantum cascade diode laser. Apart from the spectral region, the cavity conditions are comparable with the present study (i.e., 53 cm cavity length, 76 m effective path length). A detection limit of 0.015 ppmv in 5 s was reported in [49]. This comparison illustrates the favourable absorption strength in the mid-IR range, where NH_3_ was excited in one of its fundamental vibrational bands; i.e., ν_2_ out-of-plane symmetric deformation.

## 4. Conclusions 

It was shown that a gain-switched frequency comb source with an FSR of 2.5 GHz can be easily coupled to a low-finesse optical cavity in off-axes configuration for the cavity-enhanced detection of NH_3_ in the near IR range around 6605 cm^−1^. The off-axis configuration facilitated the coupling of the comb to the optical cavity without locking the narrow band laser lines to the cavity mode structure at the expense of some sensitivity. The off-axis cavity coupling scheme is experimentally simple, stable and robust (insensitive to small misalignments) in comparison with other mode-stabilized comb-cavity coupling approaches. Ultimate detection sensitivity was not expected in this proof-of-concept work due to the modest cavity mirror reflectivities and comparatively small NH_3_ absorption cross sections at ∼6605 cm^−1^. A conservative detection sensitivity of ∼(3.7 ± 0.6) ppmv of NH_3_ in 20 s is reported. This detection sensitivity is significantly below the occupational health (8 h) exposure limit of ∼20 ppmv and also well below the NH_3_ combustion limits. Hence, GSFCs are suited for monitoring of NH_3_ in industrial environments when used in conjunction with a robust off-axis cavity approach. Future work will focus on significantly more compact custom-designed photonically integrated combs (with even smaller FSR) [16,50] and on improving the detection resolution and speed through a dual comb approach [10]. Detection sensitivities at low ppbv levels are expected by employing higher finesse optical cavities. Studies with other target species in more favourable spectral regions are also planned.

## Supplementary Materials

The following are available online at https://www.mdpi.com/1424-8220/19/23/5217/s1, Figure S1: Measurements of NH_3_ absorption spectra for three different mixing ratios in 1003 mbar of air. Figure S2: HITRAN simulation of an absorption spectrum (resolution of 0.15 cm^−1^) of NH_3_ at a mixing ratio of 3.7 ppmv and H_2_O at a relative humidity (RH) of 90% in air at 1 atm. Table S1: Absorption strengths and their relative uncertainties of NH_3_ lines between 6604 and 6607 cm^−1^ and a list of some common gases that also exhibit absorption features in this wavenumber region.
