# Supplementary material for: Off-Axis Cavity-Enhanced Absorption Spectroscopy of 14NH3 in Air Using a Gain-Switched Frequency Comb at 1.514 μm
Source: Sensors (Basel). 2019 Nov 28;19(23):5217. doi: 10.3390/s19235217 (PMC6928754; doi:10.3390/s19235217)
Supplement: Supplementary file 1 [file sensors-19-05217-s001.docx]

**Supplementary Material**

Chandran et al., Off-axis cavity enhanced absorption spectroscopy of ^14^NH_3_ in air using a gain-switched frequency comb at 1.514 µm, Sensors MDPI.

**Table S1.** Absorption strengths and their relative uncertainties of NH_3_ lines between 6604 and 6607 cm^−1^ and a list of some common gases that also exhibit absorption features in this wavenumber region. The data in columns 2–5 was taken from Ref. [26].

| **Molecule** | **No. of Lines between 6604 and 6607 cm^−1^** | **Max Absorption Strength, *S*_max_,**  **in that Region**  **[cm/molecule]** | **Relative Uncertainty**  **%** | **Transition Wavenumber**  **[cm^−1^]** |
| --- | --- | --- | --- | --- |
| NH_3_ | 68 | 2.38 × 10^−21^  3.31 × 10^-21^  1.45 × 10^−21^  3.61 × 10^−21^  3.15 × 10^−21^ | 5 < ΔS < 10  5 < ΔS < 10  2 < ΔS < 5  5 < ΔS < 10  5 < ΔS < 10 | 6604.728  6605.104  6605.190  6605.609  6605.652 |
| CO_2_ | 50 | 5.16 × 10^−28^ |  | 6606.8503 |
| OCS | 57 | 7.01 × 10^−26^ |  | 6606.4128 |
| H_2_O | 33 | 1.06 × 10^−25^ |  | 6605.9890 |
| H_2_S | 7 | 1.46 × 10^−25^ |  | 6606.4042 |
| CH_4_ | 76 | 1.53 × 10^−25^  3.61 × 10^−25^ |  | 6604.0564  6604.0770 |
| C_2_H_2_ | 14 | 2.29 × 10^−21^ |  | 6605.1401 |





**Figure S1.** Measurements of NH_3_ absorption spectra for three different mixing ratios in 1003 mbar of air. The measurement conditions were the same as for the spectrum in Figure 4 shown in the main text. Insert: Linearly increasing absorption coefficient in the maxima of the two strongest bands in this region. The graph illustrates the reproducibility and linearity of the experimental approach.





**Figure S2.** HITRAN simulation of an absorption spectrum (resolution of 0.15 cm^−1^) of NH_3_ at a mixing ratio of
3.7 ppmv and H_2_O at a relative humidity (RH) of 90% in air at 1 atm. The feature at 6605.6 cm^−1^ and the background under the two main water bands are due to NH_3_ absorption at the mixing ratio stated. Through a fit of Equation (2) to the spectrum the outlined approach is still selective to NH_3_.
